# Psychological predictors of competitive levels in badminton athletes: a gender-stratified analysis

**DOI:** 10.3389/fpsyg.2025.1698010

**Published:** 2025-12-11

**Authors:** Yu Wang, Ziyan Li, Xianyan Xie, Gaoyuan Yang, Tao Chen, Huiguo Wang

**Affiliations:** 1 School of Physical Education, Guangzhou Sport University, Guangzhou, Guangdong, China; 2 School of Sport and Health, Guangzhou Sport University, Guangzhou, Guangdong, China; 3 National Academy of Badminton, Guangzhou Sport University, Guangzhou, Guangdong, China

**Keywords:** badminton athletes, character traits, competitive level, ordered logistic regression, gender differences

## Abstract

**Introduction:**

Character traits are thought to influence competitive success in racket sports, yet their predictive value for competitive level in badminton and potential gender-specific pathways remain unclear. This study aimed to investigate the predictive role of character traits on competitive level in badminton athletes, with a particular focus on gender-specific pathways.

**Methods:**

In this cross-sectional study, 392 Chinese collegiate badminton athletes (198 men and 194 women) completed the WT Character Inventory for Athletes (WTCAIA). Competitive levels (Unranked, Level 2, and Level 1) were used as an ordinal outcome. Gender-stratified ordered logistic regression models were fitted, adjusting for age and years of training. All models met the proportional odds assumption (*p* > 0.05).

**Results:**

For women (Nagelkerke *R^2^* = 0.276), resilience (*OR* = 1.026, *p* = 0.022) and flexibility (*OR* = 1.035, *p* = 0.002) were significant positive predictors of higher competitive level, whereas sociability (*OR* = 0.976, *p* = 0.031) was a negative predictor. For men (Nagelkerke R² = 0.408), resilience (*OR* = 1.073, *p* = 0.001) and emotionality (*OR* = 1.049, p = 0.026) were positive predictors, while sociability (*OR* = 0.962, *p* = 0.033) was negative. Resilience and sociability consistently predicted competitive levels across genders, whereas flexibility and emotionality showed gender-specific effects.

**Discussion:**

The higher explanatory power of the models in men suggests structural gender differences in the underlying psychological dynamics. These findings provide empirical evidence for integrating character-based assessments into badminton talent identification and for guiding the development of tailored psychological training strategies that support athlete development.

## 1 Introduction

In competitive sports, psychological factors are widely recognized as critical variables associated with athletes’ performance and developmental trajectories (Reinebo et al., 2024; Tossici et al., 2024; Tamminen et al., 2021). Among these factors, character traits—relatively stable yet moderately malleable components of psychological functioning—have attracted increasing attention in recent years for their role in predicting athletic performance (Lochbaum et al., 2022). Although previous research has examined the potential predictive value of character traits for athletes’ competitive levels (Garrido-Muñoz et al., 2024), the underlying mechanisms and modeling pathways remain insufficiently explored (Shuai et al., 2023; Allen and Laborde, 2014). In particular, effective predictive models are still lacking in sport-specific contexts (Williamson et al., 2024). Therefore, identifying key character variables and developing theoretically grounded, mechanism-based models for predicting athletes’ competitive levels has become an urgent task in sport psychology (Wang et al., 2025).

In competitive settings, traits such as resilience, emotionality, and flexibility are widely regarded as core psychological resources directly related to performance (Fletcher and Sarkar, 2012; Tamminen et al., 2024). According to trait activation theory, the behavioral expression of a trait depends on the activation of relevant situational cues (Tett et al., 2021). In badminton, high-pressure, fast-paced, and dynamic competitive environments provide precisely these cues for activating resilience, flexibility, and emotionality. Meanwhile, self-regulation theory emphasizes that athletes striving toward long-term competitive goals must regulate emotions, sustain motivation, and monitor behaviors to maintain performance consistency and psychological equilibrium (Tamminen et al., 2021). Athletes with high resilience are more likely to sustain training engagement and stable performance following injury or defeat; those with high emotional stability are better able to maintain composure during critical points; and those with high flexibility can rapidly adjust tactical responses to opponents’ changing strategies (Fletcher and Sarkar, 2012). Together, these theories elucidate the potential mechanisms through which different character traits may be associated with progression to higher competitive levels.

Although previous studies have investigated the relationship between character traits and competitive levels, the majority of these studies have relied on mixed-gender or overall samples, thereby overlooking systematic gender differences in the structure and mechanisms of these traits (Solli et al., 2024). Moreover, existing research has predominantly used general personality measures such as the NEO-PI-R and BFI. While these instruments demonstrate high reliability in general populations, their lack of sport-specific sensitivity limits their ability to capture key traits expressed in competitive contexts (Waleriańczyk and Stolarski, 2021).

Gender differences in sport psychology are well-documented, reflecting distinct motivational, emotional, and self-regulatory profiles between male and female athletes. Male athletes tend to show stronger achievement- or dominance-oriented motivation and higher self-efficacy in competitive settings, whereas female athletes often exhibit stronger relational orientation and greater use of emotional regulation and cognitive reappraisal strategies (Allen and Laborde, 2014; Hanrahan and Cerin, 2009; Millán-Sánchez et al., 2023). These distinct profiles may shape how character traits are activated and expressed in sport contexts, thereby justifying the gender-stratified analytical approach of this study.

To address these limitations, the present study employed the WT Character Inventory for Athletes (WTCAIA; Yao et al., 1995; Qi, 2010), an assessment tool developed specifically for sport contexts. This inventory focuses on “character” rather than the broader construct of “personality,” aiming to capture stable character traits within core competitive scenarios such as training, competition, and cooperation. The WTCAIA assesses eight dimensions closely related to athletic performance, namely resilience, flexibility, self-control, sociability, emotionality, rationality, excitability, and ambition. Compared to generic personality inventories, the WTCAIA offers higher ecological validity and task relevance by more effectively capturing athlete traits in competitive settings. Recent studies involving athlete samples have applied the WTCAIA in competitive contexts and found acceptable subscale reliability and trait differentiation, supporting its continued use in sport-specific research (Zhan, 2016; Ye et al., 2021). Furthermore, we utilized a gender-stratified ordinal logistic regression model (Fullerton, 2009) to precisely delineate the predictive pathways of these traits across different genders, while controlling for demographic variables and training years. This methodological approach is not only well-suited for the ordinal nature of competitive levels (unranked < Level 2 < Level 1) but also enhances the explanatory power of the gender-sensitive analysis.

In this study, competitive levels refer to China’s nationally standardized Athlete Technical Grade in badminton: Unranked, Level 2, and Level 1—granted based on the official Athlete Technical Grade Standards (Badminton) issued by General Administration of Sport of China (2024). Grades are awarded based on athletes’ placements in officially sanctioned tournaments (e.g., national championships, elite collegiate or provincial competitions) and verified through institutional or association registries (Zhu, 2024). Accordingly, the competitive level was modeled as an ordinal variable coded as follows: 0 = Unranked, 1 = Level 2, and 2 = Level 1, with higher values indicating higher competitive attainment.

Based on this background, the present study targeted Chinese collegiate badminton athletes with the aims (1) to describe the character traits of athletes across competitive levels and gender groups and (2) to construct a character-based predictive model of competitive levels and examining gender differences in its predictive effects. The findings aim to provide empirical evidence for scientific talent identification, psychological assessment, and gender-sensitive interventions in badminton. Therefore, this study employed a gender-stratified ordinal logistic regression to examine how specific character traits predict competitive levels, thereby linking the theoretical rationale to empirical validation.

## 2 Materials and methods

### 2.1 Study design and ethical statement

This study employed a cross-sectional survey design, selected for its effectiveness in capturing a “snapshot” of the associations between character traits and existing competitive levels among athletes. In this context, the term “predict” is used in a statistical sense—to identify significant associative variables in regression models—and does not imply a causal relationship. Participants were recruited via purposive sampling between May and June 2025 from several universities in Guangdong Province renowned for their competitive badminton programs, including Guangzhou Sport University, South China Normal University, and Jinan University. The purposive sampling strategy targeted members of university varsity teams to ensure adequate representation across competitive levels (Unranked, Level 2, and Level 1) and balanced gender distribution. Eligible participants were required to possess a formal, systematic training background in badminton. Competitive level classification (Unranked, Level 2, and Level 1) was defined in accordance with the Athlete Technical Grade Standards (Badminton) issued by General Administration of Sport of China (2024), with verification of certified grades when applicable.

Inclusion criteria are as follows: (1) currently enrolled university students aged 18–25 years; (2) at least 3 years of systematic, sport-specific badminton training; and (3) no history of severe psychological disorders or cognitive impairments. Exclusion criteria are as follows: (1) questionnaires with substantial missing data or logical inconsistencies; and (2) training interruption within the preceding 3 months due to injury. All participants provided electronic informed consent before completing the survey. The study protocol was approved by the Institutional Review Board of Guangzhou Sport University (Approval No. 2025LCLL-065). It was conducted in accordance with the ethical principles outlined in the Declaration of Helsinki.

### 2.2 Participants and grouping

A total of 415 questionnaires were distributed, yielding 392 valid responses (response rate = 94.5%). Participants’ competitive levels were determined according to the Athlete Technical Grade Standards issued by the General Administration of Sport of China, based on self-reports and verified by their coaches. They were categorized into three levels: Level 1 athletes (*n* = 127), Level 2 athletes (*n* = 130), and unranked athletes (*n* = 135). Based on self-reported gender, participants were divided into male (*n* = 198) and female (*n* = 194) groups. To examine gender heterogeneity, all statistical analyses were stratified by gender.

### 2.3 Demographics and training background

A self-developed questionnaire was used to collect data on participants’ gender, year of birth, years of sport-specific training, and current highest technical grade.

### 2.4 Character trait assessment

Character traits were assessed using the WTCAIA (Yao et al., 1995; Qi, 2010), an instrument specifically designed for the Chinese competitive sport context. This inventory is tailored for athletes and demonstrates high cultural relevance and sport-specific validity. It comprises eight core dimensions:

Resilience: the ability to maintain effort and goal orientation amid adversity, pressure, or failure.

Flexibility: the ability to adapt thoughts, tactics, and behaviors in response to changing environments or opponents.

Self-control: the capacity to manage impulses, maintain focus, and adhere to tactical plans under pressure.

Sociability: proactivity and adaptability in interpersonal interactions within training and team settings.

Emotionality: the stability of emotional states and the capacity for emotion regulation, where a higher score indicates greater emotional stability.

Rationality: the ability to engage in logical, analytical, and objective decision-making.

Excitability: the level of psychological activation and proactive engagement in training and competition.

Ambition: the drive to pursue athletic excellence and the motivation to compete.

The WTCAIA contains 72 items: eight per dimension, a seven-item lie scale, and one non-scored introductory item. Responses are recorded on a 5-point Likert scale (1 = never to 5 = always). Reverse-coded items were rescored (recoded score = 6 − original score) before analysis. Raw scores were adjusted for social desirability bias using the lie scale scores, following the procedure in the official manual’s “Original Score Correction Method Table.” Adjusted scores were converted to T-scores (*M* = 50, *SD* = 10) based on gender-specific norms to ensure comparability. Reported internal consistency (Cronbach’s *α*) for the dimensions generally ranges from 0.60 to 0.80, and factor analysis has confirmed strong structural validity (factor loadings > 0.90 on primary factors with low cross-loadings; Yao et al., 1995; Qi, 2010).

### 2.5 Statistical analysis

All analyses were conducted using IBM SPSS Statistics 26.0, with the significance level set at *p* = 0.05. Descriptive statistics were computed for the T-scores of the eight-character traits, with continuous variables expressed as mean ± standard deviation (*M* ± *SD*). Multicollinearity was assessed using the variance inflation factor (VIF) and tolerance, with VIF < 5 and tolerance > 0.20 indicating no severe multicollinearity. Given the ordinal nature of the dependent variable, gender-stratified ordinal logistic regression models were constructed. The dependent variable, competitive level, was modeled as a three-level ordinal outcome, coded as 0 = Unranked, 1 = Level 2, and 2 = Level 1. The T-scores of the eight traits were entered as independent variables, with age and years of training included as covariates. Model fit was evaluated using the −2 log likelihood (−2LL), chi-square goodness-of-fit, Nagelkerke *R^2^*, Akaike’s information criterion (AIC), and Bayesian information criterion (BIC). The proportional odds assumption was tested via the test of parallel lines. The results of regression are reported with coefficients (*B*), standard errors (*SE*), Wald statistics, *p*-values, odds ratios (*OR*), and 95% confidence intervals (CI).

## 3 Results

### 3.1 Descriptive statistics of character traits by gender and competitive level

A total of 392 badminton athletes (198 men and 194 women) were included in this study, comprising 127 Level 1 athletes (32.4%), 130 Level 2 athletes (33.2%), and 135 unranked athletes (34.4%), with a relatively balanced distribution of competitive levels. Table 1 presents the mean (*M*) and standard deviation (*SD*) of the eight-character traits across gender and competitive level groups.

**Table 1:** Descriptive statistics of psychological traits by competitive level and gender.

Gender	Trait	Unranked (*M* ± *SD*)	Level 2 (*M* ± *SD*)	Level 1 (*M* ± *SD*)
Male	Resilience	56 ± 8.56	62 ± 10.44	70 ± 5.51
(*n* = 198)	Sociability	69 ± 9.43	65 ± 10.59	60 ± 4.56
	Flexibility	59 ± 9.10	63 ± 9.63	69 ± 4.67
	Excitability	57 ± 8.90	62 ± 10.96	67 ± 5.51
	Rationality	56 ± 8.23	58 ± 10.77	64 ± 4.79
	Self-control	73 ± 8.23	71 ± 9.90	70 ± 8.09
	Emotionality	67 ± 9.13	71 ± 8.23	70 ± 8.80
	Ambition	71 ± 9.30	72 ± 9.86	69 ± 8.22
*n* (%)		*n* = 65 (32.82%)	*n* = 69 (34.84%)	*n* = 64 (32.32%)
Female	Resilience	61 ± 13.11	64 ± 13.34	68 ± 12.63
(*n* = 194)	Sociability	71 ± 12.99	64 ± 14.39	64 ± 10.68
	Flexibility	60 ± 12.60	66 ± 12.63	69 ± 13.46
	Self-control	64 ± 8.16	67 ± 8.76	70 ± 8.56
	Excitability	64 ± 9.18	66 ± 8.70	70 ± 7.86
	Rationality	64 ± 8.55	65 ± 9.11	68 ± 9.00
	Emotionality	65 ± 8.71	67 ± 8.69	69 ± 8.51
	Ambition	64 ± 8.13	65 ± 8.87	69 ± 8.74
*n* (%)		*n* = 70 (36.08%)	*n* = 61 (31.44%)	*n* = 63 (32.47%)

Values are presented as *M* ± *SD* and expressed as T-scores. Percentages indicate the proportion within each gender group.

Among male athletes, the unranked group had lower scores in resilience (56.0 ± 8.56), flexibility (59.0 ± 9.10), and emotionality (67.0 ± 9.13) compared with the Level 1 group (70.0 ± 5.51, 69.0 ± 4.67, and 70.0 ± 8.80, respectively). In sociability, the Level 1 group (60.0 ± 4.56) scored lower than the unranked group (69.0 ± 9.43). Among female athletes, the unranked group had lower scores in resilience (61.0 ± 13.11), flexibility (60.0 ± 12.60), and self-control (64.0 ± 8.16) compared with the Level 1 group (68.0 ± 12.63, 69.0 ± 13.46, and 70.0 ± 8.56, respectively). In sociability, the Level 1 group (64.0 ± 10.68) scored lower than the unranked group (71.0 ± 12.99).

In summary, a comparison between the highest (Level 1) and lowest (unranked) competitive groups revealed distinct patterns. Level 1 male athletes exhibited higher resilience, flexibility, and emotionality scores than the unranked group. Similarly, Level 1 female athletes showed higher resilience, flexibility, and self-control than the unranked female group. Conversely, for both genders, the Level 1 group reported lower sociability than the unranked group.

### 3.2 Predictive results of character traits on competitive level in female badminton athletes

As shown in Table 2, the ordinal logistic regression model for female athletes was statistically significant, *χ^2^*(8) = 54.670, *p* < 0.001, indicating that the predictors effectively distinguished competitive levels. The model showed good fit (Nagelkerke *R^2^* = 0.276; AIC = 388.908; BIC = 418.311), and the test of parallel lines was non-significant [*χ^2^*(8) = 4.903, *p* = 0.768], meeting the assumptions of ordinal logistic regression. Multicollinearity diagnostics indicated that all predictors had VIF < 1.22 and tolerance > 0.82, suggesting no severe multicollinearity.

**Table 2:** Model fit indices and proportional odds assumption tests for gender-stratified ordered logistic regression models (N = 392).

Gender (n)	Model	−2Log Likelihood (−2LL)	*χ^2^*	*df*	*p-value*	AIC	BIC	Nagelkerke *R*^2^	PLT *χ^2^*	*df*	*p*
Female (194)	Intercept-only	425.578				427.58	430.85				
	Full	370.908	54.670	8	<0.001	388.91	418.31	0.276	4.903	8	0.768
Male (198)	Intercept-only	434.840				438.84	445.45				
	Full	344.406	90.433	8	<0.001	364.41	397.24	0.408	11.416	8	0.179

PLT, Parallel Line Test for proportional-odds assumption; −2LL, Minus Two Log-Likelihood; AIC, Akaike Information Criterion; BIC, Bayesian Information Criterion. Non-significant *p*-values (*p* > 0.05) indicate the assumption is met.

After controlling for age and years of training, resilience (*OR* = 1.026, 95% *CI*: 1.004, 1.048, *p* = 0.022) and flexibility (*OR* = 1.035, 95% *CI*: 1.013, 1.058, *p* = 0.002) were significant positive predictors of competitive levels, while sociability (*OR* = 0.976, 95% *CI*: 0.955, 0.998, *p* = 0.031) was a significant negative predictor. Furthermore, excitability (*OR* = 1.034, 95% *CI*: 0.999, 1.043, *p* = 0.054) and emotionality (*OR* = 1.031, 95% *CI*: 0.997, 1.066, *p* = 0.079) exhibited marginally significant positive trends (Table 3).

**Table 3:** Ordered logistic regression results for a female badminton athlete.

Variable	*B*	*SE*	Wald *χ^2^*	*p-value*	*OR*	95% confidence interval (lower–upper)
Resilience	0.025	0.011	5.220	0.022^*^	1.026	1.004–1.049
Sociability	−0.024	0.011	4.667	0.031^*^	0.976	0.955–0.998
Flexibility	0.034	0.011	9.514	0.002^**^	1.035	1.013–1.058
Self-control	0.028	0.017	2.774	0.096	1.029	0.995–1.064
Excitability	0.033	0.017	3.706	0.054	1.034	0.999–1.070
Rationality	0.023	0.016	2.093	0.148	1.024	0.992–1.057
Emotionality	0.030	0.017	3.088	0.079	1.031	0.997–1.066
Ambition	0.025	0.017	2.191	0.139	1.026	0.992–1.061

Competitive level was coded in ascending order (0 = Unranked < 1 = Level 2 < 2 = Level 1), such that positive coefficients indicate a greater likelihood of attaining a higher competitive level. B = regression coefficient; SE = standard error; Wald *χ*^2^ = Wald chi-square statistic; OR = odds ratio; CI = confidence interval (Lower–Upper); Nagelkerke *R*^2^ = 0.276; all VIFs ranged between 1.02 and 1.22. ^*^p < 0.05, ^**^p < 0.01.

### 3.3 Predictive results of character traits on the competitive level in male badminton athletes

As shown in Table 2, the ordinal logistic regression model for male athletes was statistically significant, *χ^2^*(8) = 90.433, *p* < 0.001, indicating that the predictors effectively distinguished competitive levels. The model demonstrated good fit (Nagelkerke *R^2^* = 0.408; AIC = 364.406; BIC = 397.238), and the test of parallel lines was non-significant [*χ^2^*(8) = 11.416, *p* = 0.179], meeting the assumptions of ordinal logistic regression. Multicollinearity diagnostics indicated that all predictors had VIF < 1.22 and tolerance > 0.82, suggesting no severe multicollinearity.

After controlling for age and years of training, resilience (*OR* = 1.073, 95% *CI*: 1.030, 1.118, *p* = 0.001) and emotionality (*OR* = 1.049, 95% *CI*: 1.006, 1.094, *p* = 0.026) were significant positive predictors of competitive level, while sociability (*OR* = 0.962, 95% *CI*: 0.929, 0.997, *p* = 0.033) was a significant negative predictor. The remaining traits did not reach statistical or marginal significance (Table 4).

**Table 4:** Ordered logistic regression results for male badminton athletes.

Variable	*B*	*SE*	Wald *χ^2^*	*p-value*	OR	95% confidence interval (lower–upper)
Resilience	0.070	0.021	11.291	0.001^**^	1.073	1.030–1.118
Sociability	−0.038	0.018	4.563	0.033^*^	0.962	0.929–0.997
Flexibility	0.033	0.021	2.480	0.115	1.034	0.992–1.077
Excitability	0.026	0.018	2.002	0.157	1.026	0.990–1.063
Rationality	0.020	0.019	1.051	0.305	1.020	0.982–1.060
Self-control	−0.037	0.024	2.336	0.126	0.964	0.919–1.012
Emotionality	0.048	0.021	4.966	0.026^*^	1.049	1.006–1.094
Ambition	−0.003	0.024	0.020	0.888	0.997	0.951–1.044

Competitive level was coded in ascending order (0 = Unranked < 1 = Level 2 < 2 = Level 1), such that positive coefficients indicate a greater likelihood of attaining a higher competitive level. B = regression coefficient; SE = standard error; Wald *χ*^2^ = Wald chi-square statistic; OR = odds ratio; CI = confidence interval (Lower–Upper); Nagelkerke *R*^2^ = 0.408; all VIFs ranged between 1.02 and 1.22. ^*^p < 0.05, ^**^p < 0.01.

## 4 Discussion

Before interpreting the specific findings, it is important to acknowledge the overall explanatory power of our models. The Nagelkerke *R*^2^ values of 0.276 for women and 0.408 for men, while statistically significant, indicate that character traits account for a modest portion of the variance in competitive level. This underscores that psychological attributes are important yet operate within a broader multifactorial system encompassing physical conditioning, technical skill, tactical acumen, and coaching support—factors not measured in this study. The following discussion, therefore, focuses on the associative patterns observed, recognizing that they represent one dimension of a more complex performance framework.

Using an ordinal logistic regression framework, this study examined the predictive associations of character traits with competitive level in badminton athletes and explored gender-specific pathways through gender-stratified modeling. The results indicated that resilience and sociability consistently predicted competitive levels across genders, whereas flexibility and emotionality exhibited gender-specific predictive patterns.

### 4.1 Resilience as a significant positive predictor of competitive level

Resilience emerged as a significant positive predictor for both male (*OR* = 1.073, 95% *CI*: 1.030, 1.118) and female (*OR* = 1.026, 95% *CI*: 1.004, 1.048) athletes. This finding aligns with the international consensus that psychological resilience and perseverance constitute core psychological capital for elite performance (Gupta and McCarthy, 2022). Within the framework of self-regulation theory, resilience can be viewed as a central component of athletes’ self-regulatory systems, integrating goal setting, motivational maintenance, and emotional regulation processes. This capacity is associated with sustained engagement and performance stability even in the face of major setbacks, injuries, or monotonous training cycles (Den Hartigh et al., 2024; Rogowska and Tataruch, 2024). This sustained engagement and behavioral consistency in adversity likely underpin its stable predictive value across genders.

### 4.2 Sociability as a significant negative predictor of competitive level

Conversely, in both women (*OR* = 0.976) and men (*OR* = 0.962), sociability showed a small but statistically significant negative association with the competitive level. This counterintuitive relationship may be interpreted through attentional-resource models of high-pressure performance. Elite badminton represents an open-skill sport characterized by continuous perceptual–cognitive processing and rapid decision-making cycles. Under such cognitively demanding conditions, diverting limited attentional resources toward task-irrelevant social cues may compromise tactical execution and shot accuracy. Previous research on anxiety and attention under competitive stress indicates that exposure to social-evaluative or threat-related stimuli biases attentional focus, shortens quiet-eye duration, and disrupts goal-directed control, ultimately impairing perceptual-motor coordination (Nieuwenhuys and Oudejans, 2012; Nieuwenhuys and Oudejans, 2017). Athletes with high sociability are more sensitive to interpersonal cues and may divert cognitive resources to crowd reactions, opponents’ body language, or self-evaluative thoughts. This attentional diversion competes with on-task processing and decision accuracy during prolonged, complex rallies. Consistent with this reasoning, studies of open-skill athletes have demonstrated greater executive-control demands and performance costs under high-pressure conditions when attentional focus becomes fragmented (Koch and Krenn, 2021; Heilmann et al., 2022). Therefore, the negative association observed in this study likely reflects an attentional trade-off in cognitively demanding environments rather than a maladaptive personality per se.

### 4.3 Gender-differentiated predictive roles of flexibility and emotionality

Flexibility emerged as a positive predictor among female athletes (*OR* = 1.035). The WTCAIA defines flexibility as an athlete’s ability to adjust quickly to changes in training or match conditions and to modify tactics in response to opponents’ strategies—that is, cognitive openness and strategic adaptability (Qi, 2010). Match-analysis studies indicate that women’s elite badminton often involves longer rallies and more frequent tactical exchanges than men’s matches, thereby increasing the importance of in-rally re-planning (Gomez et al., 2020; Iizuka et al., 2020). In line with trait activation theory (Tett et al., 2021), such situational characteristics are likely to trigger the behavioral expression of flexibility, enabling athletes to switch more efficiently between offensive and defensive intentions, exploit emergent weaknesses, and regulate momentum throughout extended rallies. Evidence from open-skill sports further demonstrates that athletes in these environments exhibit enhanced executive control and cognitive flexibility relative to those in closed-skill disciplines, linking flexibility to performance advantages when continuous perception–action adjustments are required (Koch and Krenn, 2021; Heilmann et al., 2022). Collectively, these points help explain the gender-specific predictive value of flexibility observed in this study.

Emotionality emerged as a significant positive predictor among male athletes (*OR* = 1.049, 95% *CI*: 1.006, 1.094). In the WTCAIA, the scale for emotionality assesses an individual’s capacity to maintain emotional balance and control impulsive behaviors under pressure, where a higher score indicates greater stability. Men’s badminton matches are typically accompanied by faster shuttle speeds, greater rally intensity, and heightened psychological and physical demands (Green et al., 2023). Emotional fluctuations may be linked to reduced technical consistency and tactical coherence, and empirical evidence indicates that athletes with poor emotion regulation and high impulsivity tend to exhibit higher error rates (Hufton et al., 2024). Furthermore, emotion regulation frameworks suggest that depletion of emotional control resources may interfere with rhythm maintenance and tactical execution stability, thereby increasing variability in competitive performance (Millán-Sánchez et al., 2023). This mechanism aligns with the predictions of the “emotional stability–attentional allocation–performance quality” pathway model (Tamminen et al., 2025), which posits that emotional stability is thought to support technical consistency and performance stability in key rallies by facilitating optimal attentional resource allocation (Rogowska and Tataruch, 2024). This pattern has also been validated in other high-intensity racquet sports, where emotionally stable athletes generally maintain higher performance stability during critical points (Klatt et al., 2021).

### 4.4 Theoretical interpretation and boundary conditions of non-significant character traits

In the gender-stratified models of this study, ambition, self-control, and rationality did not significantly predict competitive level. This finding suggests that the influence of certain character traits may depend on contextual congruence or operate through indirect pathways, rather than exerting a direct effect on competitive advancement across all competition scenarios (Tett et al., 2021).

Ambition reflects achievement motivation, a sense of responsibility, and sustained effort, attributes that typically manifest as advantages in long-term training adherence and career development trajectories. However, competitive level classification in China is predominantly based on short-term performance outcomes; thus, the cumulative advantages of ambition are difficult to capture within the cross-sectional design of the present study (Williamson et al., 2024; Kovács and Szakál, 2024).

Self-control reflects the ability to inhibit impulses inconsistent with tactical objectives, maintain task focus, and adhere to long-term training plans. Although these abilities form the foundation of elite performance, the generally high and homogeneous levels of self-control in elite athlete samples can create a “ceiling effect,” thereby reducing their discriminatory power in the model (Englert, 2025; Zentgraf et al., 2024).

Rationality represents the capacity for logical analysis and objective judgment in complex situations. This trait may hold greater value in pre-competition tactical preparation and long-term strategic planning. However, the fast-paced, real-time decision-making demands of badminton matches make it difficult for rationality advantages to translate into immediate performance gains, and these effects may be overshadowed by traits with greater reactivity or flexibility (Wu et al., 2024; Iizuka et al., 2020).

For female athletes, the marginal significance of emotionality (*p* = 0.079) and excitability (*p* = 0.054) suggests that these traits may exert a positive role in relation to competitive levels under certain conditions. For example, athletes with high excitability may demonstrate faster response initiation and offensive decision-making in high-tempo rallies. A high score on emotionality may provide women with psychological stability similar to that observed in men, although this effect did not reach statistical significance in the current sample and classification framework (Millán-Sánchez et al., 2023; Gershgoren et al., 2023).

### 4.5 Practical implications and applied recommendations for predicting competitive level

The gender-stratified analysis in this study revealed that resilience and sociability were stable predictors across both male and female athletes, whereas flexibility and emotionality exhibited gender-specific effects. These results indicate that psychological traits are not only important correlates of performance but also essential components in talent identification and promotion assessments (Piepiora and Piepiora, 2021). Given the modest explanatory power of the models, these associations should be interpreted as indicative rather than causal. Incorporating psychological assessment alongside technical and physical evaluations may help address the limitations of relying solely on competitive results and could improve the precision of identifying athletes with long-term developmental potential (Höner et al., 2021). For example, resilience, considered a core psychological capital of elite performance, is closely linked to persistence toward long-term goals and the ability to withstand setbacks (Gupta and McCarthy, 2022). The negative association of sociability with competitive levels in high cognitive-load contexts suggests that excessive extroversion may be associated with reduced focus and tactical execution efficiency (Laborde et al., 2016). The positive predictive role of flexibility in women underscores the need to evaluate tactical adaptability and strategy-switching capacity in female events (Ren et al., 2025). Conversely, the positive role of high scores on the emotionality scale in men highlights the importance of maintaining psychological stability under high-pressure conditions (Millán-Sánchez et al., 2023). These findings provide empirical support for integrating psychological dimensions into competitive-level promotion systems.

In practical application, psychological intervention strategies should be gender-sensitive and context-specific to support performance enhancement (Reinebo et al., 2024). Resilience can be strengthened through adversity simulations, delayed feedback, and setback-challenge training (Sarkar and Fletcher, 2014); flexibility can be developed via variable-speed multi-shuttle drills, tactical shift simulations, and situational transition tasks (Başoğlu et al., 2025). Emotional stability can be enhanced through evidence-based interventions, such as cognitive reappraisal training, biofeedback techniques, and simulated high-pressure match scenarios (Corrado et al., 2024; Meng et al., 2024); and for athletes with high sociability, attention control exercises and pre-competition focus interventions can be used to minimize distraction from irrelevant social stimuli (Tamminen et al., 2021). The systematic implementation of these strategies may offer potential pathways for sustainable competitive improvement.

### 4.6 Limitations and directions for future research

Several limitations should be noted in this study. First, the cross-sectional design, while revealing significant associations between character traits and competitive levels, precludes causal inference. Future research should employ longitudinal tracking or randomized controlled trials (RCTs) to verify the causal pathways of key traits in competitive advancement. Second, the measurement approach relied on self-reported assessments, which may be subject to social desirability and self-perception biases. To enhance objectivity and ecological validity, future studies could adopt a multi-method assessment approach, incorporating coach evaluations, behavioral video analysis during competition, and physiological monitoring. A third limitation concerns the measurement instrument. The WTCAIA possesses high sport-specific ecological validity and has demonstrated good reliability and structural validity in previous research (Yao et al., 1995; Qi, 2010). Building on this foundation, future research could systematically establish its broader utility by examining its factor structure’s cross-sample stability, measurement invariance across genders, and its criterion-related validity with other psychological constructs across diverse cultural and sport contexts to consolidate its psychometric foundation. Finally, the sample was limited to Chinese collegiate badminton athletes. Cultural and sport-specific factors may limit the generalizability of the findings. Future work should include cross-cultural and cross-sport comparative studies to examine the universality and boundary conditions of the observed effects.

## 5 Conclusion

Based on gender-stratified ordinal logistic regression models, this study’s findings suggest stable, common predictive associations of resilience and sociability at the competitive level in both male and female badminton athletes. The results also revealed gender-specific effects, with flexibility emerging as a key predictor in female athletes and emotionality in male athletes. The findings indicated that incorporating key character traits into athlete selection and promotion assessment systems, together with gender-sensitive psychological intervention strategies, can improve the precision of identifying potential athletes and improving the scientific basis of their competitive development pathways. This study provides new empirical evidence on the gender-differentiated associations between sport-specific character traits and competitive levels, enriches the theoretical framework of sport psychology, and offers practical, actionable approaches for the scientific selection and psychological training optimization of elite badminton athletes.

## Data Availability

The original contributions presented in the study are included in the article/supplementary material, further inquiries can be directed to the corresponding authors.
